# Supplementary material for: Prevalence of Occult Central Lymph Node Metastasis by Tumor Size in Papillary Thyroid Carcinoma: A Systematic Review and Meta-Analysis
Source: Curr Oncol. 2023 Aug 2;30(8):7335–50. doi: 10.3390/curroncol30080532 (PMC10453273; doi:10.3390/curroncol30080532)
Supplement: Supplementary file 1 [file curroncol-30-00532-s001.zip › Supplemental Figure 1 Baujat Leave one out.pdf]

Supplemental Figure S1.

Baujat plots and leave-one-out statistical outputs

Less than 5mm

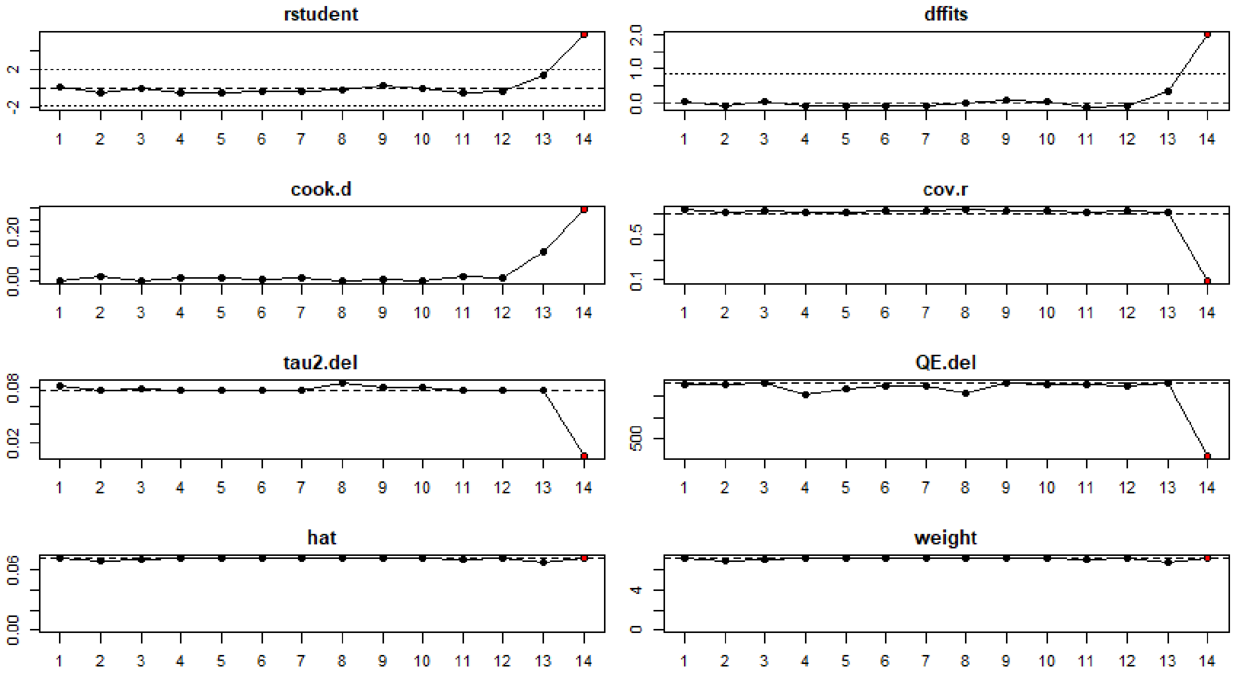

	rstudent	dffits	cook.d	cov.r	tau2.del	QE.del	hat	weight	dfbs	inf
1	0.0979	0.0268	0.0008	1.1402	0.0812	1802.3588	0.0721	7.2076	0.0268	
2	-0.4680	-0.1279	0.0164	1.0759	0.0767	1808.6248	0.0695	6.9496	-0.1279	
3	0.0084	0.0022	0.0000	1.0945	0.0780	1820.7700	0.0707	7.0709	0.0022	
4	-0.4362	-0.1219	0.0148	1.0760	0.0765	1554.9362	0.0724	7.2446	-0.1219	
5	-0.4481	-0.1249	0.0157	1.0815	0.0769	1704.9657	0.0721	7.2093	-0.1249	
6	-0.3242	-0.0902	0.0082	1.0908	0.0776	1772.8179	0.0717	7.1672	-0.0902	
7	-0.3928	-0.1092	0.0120	1.0852	0.0772	1765.5174	0.0716	7.1649	-0.1092	
8	-0.1499	-0.0427	0.0020	1.1938	0.0850	1587.1703	0.0726	7.2554	-0.0427	
9	0.2394	0.0662	0.0046	1.1388	0.0811	1819.5882	0.0720	7.1962	0.0662	
10	0.0050	0.0010	0.0000	1.1215	0.0798	1799.5871	0.0719	7.1905	0.0010	
11	-0.5045	-0.1395	0.0194	1.0762	0.0766	1782.6440	0.0710	7.0982	-0.1395	
12	-0.3961	-0.1103	0.0123	1.0863	0.0773	1750.7264	0.0718	7.1833	-0.1103	
13	1.2808	0.3458	0.1204	1.0807	0.0772	1829.1770	0.0679	6.7909	0.3457	
14	5.6535	2.0101	0.2935	0.0947	0.0055	90.2390	0.0727	7.2714	1.8423	*

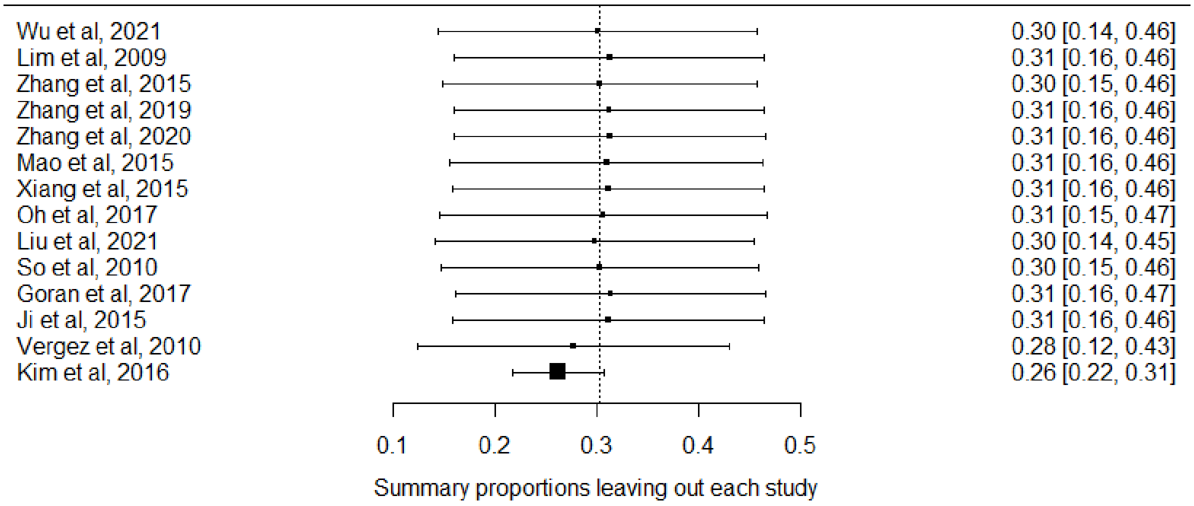


```
estimate     se     zval   pval  ci.lb  ci.ub          Q     Qp   tau2
1    0.3009 0.0797  3.7733 0.0002 0.1446 0.4572 1802.3588 0.0000 0.0812
2    0.3125 0.0775  4.0345 0.0001 0.1607 0.4643 1808.6248 0.0000 0.0767
3    0.3028 0.0781  3.8756 0.0001 0.1497 0.4559 1820.7700 0.0000 0.0780
4    0.3121 0.0775  4.0283 0.0001 0.1602 0.4639 1554.9362 0.0000 0.0765
5    0.3123 0.0777  4.0214 0.0001 0.1601 0.4645 1704.9657 0.0000 0.0769
6    0.3097 0.0780  3.9713 0.0001 0.1569 0.4626 1772.8179 0.0000 0.0776
7    0.3111 0.0778  3.9994 0.0001 0.1587 0.4636 1765.5174 0.0000 0.0772
8    0.3063 0.0816  3.7540 0.0002 0.1464 0.4662 1587.1703 0.0000 0.0850
9    0.2979 0.0797  3.7379 0.0002 0.1417 0.4541 1819.5882 0.0000 0.0811
10   0.3029 0.0791  3.8297 0.0001 0.1479 0.4579 1799.5871 0.0000 0.0798
11   0.3134 0.0775  4.0449 0.0001 0.1615 0.4652 1782.6440 0.0000 0.0766
12   0.3112 0.0778  3.9985 0.0001 0.1587 0.4638 1750.7264 0.0000 0.0773
13   0.2771 0.0776  3.5687 0.0004 0.1249 0.4292 1829.1770 0.0000 0.0772
14   0.2625 0.0230 11.4234 0.0000 0.2175 0.3075   90.2390 0.0000 0.0055
        I2       H2
1  99.3342 150.1966
2  99.3365 150.7187
3  99.3409 151.7308
4  99.2283 129.5780
5  99.2962 142.0805
6  99.3231 147.7348
7  99.3203 147.1265
8  99.2439 132.2642
9  99.3405 151.6323
10 99.3332 149.9656
11 99.3268 148.5537
12 99.3146 145.8939
13 99.3440 152.4314
14 86.7020   7.5199
```

Less than 1cm

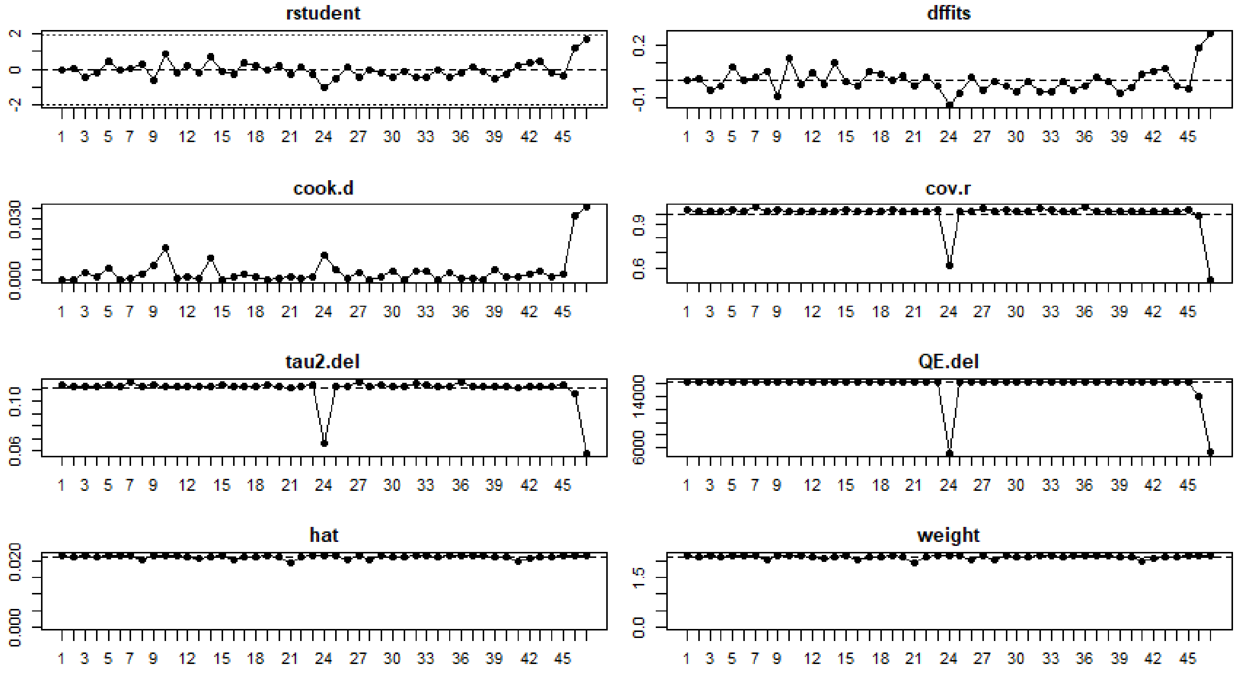

	rstudent	dffits	cook.d	cov.r	tau2.del	QE.del	hat	weight
1	0.0102	0.0015	0.0000	1.0347	0.1130	16292.4374	0.0216	2.1577
2	0.0731	0.0108	0.0001	1.0255	0.1120	16294.0800	0.0213	2.1258
3	-0.3938	-0.0584	0.0034	1.0294	0.1124	16291.0683	0.0215	2.1483
4	-0.2017	-0.0298	0.0009	1.0272	0.1121	16296.2430	0.0214	2.1379
5	0.5081	0.0755	0.0058	1.0346	0.1129	16202.0926	0.0216	2.1620
6	-0.0005	-0.0001	0.0000	1.0302	0.1125	16294.1713	0.0215	2.1500
7	0.0927	0.0137	0.0002	1.0648	0.1163	16264.9447	0.0217	2.1678
8	0.3458	0.0500	0.0025	1.0221	0.1117	16292.2683	0.0205	2.0462
9	-0.5739	-0.0853	0.0074	1.0341	0.1129	16267.4082	0.0216	2.1587
10	0.8484	0.1258	0.0159	1.0246	0.1118	16186.9295	0.0215	2.1524
11	-0.1809	-0.0268	0.0007	1.0302	0.1125	16296.3190	0.0215	2.1497
12	0.2652	0.0390	0.0015	1.0242	0.1118	16290.2261	0.0211	2.1122
13	-0.1548	-0.0227	0.0005	1.0239	0.1118	16296.4022	0.0210	2.0985
14	0.6964	0.1029	0.0106	1.0243	0.1118	16252.2026	0.0214	2.1372
15	-0.0580	-0.0087	0.0001	1.0368	0.1132	16294.8810	0.0216	2.1596
16	-0.2249	-0.0325	0.0011	1.0224	0.1117	16296.3011	0.0205	2.0490
17	0.3484	0.0515	0.0027	1.0263	0.1120	16280.5851	0.0214	2.1382
18	0.2421	0.0358	0.0013	1.0266	0.1121	16286.7382	0.0214	2.1377
19	0.0104	0.0015	0.0000	1.0350	0.1130	16292.3482	0.0216	2.1580
20	0.1879	0.0277	0.0008	1.0252	0.1119	16291.2586	0.0212	2.1243
21	-0.2203	-0.0312	0.0010	1.0209	0.1116	16296.3447	0.0197	1.9655
22	0.1047	0.0155	0.0002	1.0269	0.1121	16292.2816	0.0214	2.1379
23	-0.2222	-0.0330	0.0011	1.0376	0.1133	16295.4786	0.0216	2.1601
24	-0.9557	-0.1425	0.0120	0.6117	0.0658	5184.0109	0.0217	2.1718
25	-0.4845	-0.0718	0.0052	1.0285	0.1123	16287.1550	0.0215	2.1462
26	0.1275	0.0183	0.0003	1.0217	0.1117	16295.3734	0.0202	2.0159
27	-0.3858	-0.0575	0.0034	1.0603	0.1158	16269.8901	0.0217	2.1673
28	-0.0414	-0.0060	0.0000	1.0224	0.1117	16296.2054	0.0205	2.0487
29	-0.2007	-0.0298	0.0009	1.0375	0.1133	16295.9586	0.0216	2.1600
30	-0.4510	-0.0661	0.0044	1.0240	0.1118	16293.5675	0.0210	2.1039
31	-0.0638	-0.0094	0.0001	1.0260	0.1120	16296.0212	0.0213	2.1294
32	-0.4179	-0.0622	0.0040	1.0487	0.1145	16272.5597	0.0217	2.1654
33	-0.4316	-0.0641	0.0042	1.0345	0.1129	16284.1741	0.0216	2.1581
34	-0.0442	-0.0065	0.0000	1.0258	0.1120	16295.8486	0.0213	2.1279
35	-0.3888	-0.0576	0.0033	1.0296	0.1124	16291.1581	0.0215	2.1489
36	-0.1947	-0.0290	0.0009	1.0648	0.1163	16295.3588	0.0217	2.1676

37	0.1272	0.0189	0.0004	1.0325	0.1127	16286.3059	0.0216	2.1554
38	-0.0667	-0.0099	0.0001	1.0323	0.1127	16295.5319	0.0215	2.1542
39	-0.4789	-0.0707	0.0050	1.0264	0.1120	16290.1805	0.0214	2.1350
40	-0.2540	-0.0373	0.0014	1.0246	0.1119	16296.0205	0.0211	2.1122
41	0.2379	0.0338	0.0011	1.0210	0.1116	16294.7926	0.0198	1.9772
42	0.3619	0.0527	0.0028	1.0228	0.1117	16290.4782	0.0208	2.0779
43	0.4444	0.0653	0.0043	1.0239	0.1118	16283.2972	0.0211	2.1143
44	-0.2027	-0.0299	0.0009	1.0260	0.1120	16296.2703	0.0213	2.1289
45	-0.3425	-0.0509	0.0026	1.0345	0.1129	16290.8863	0.0216	2.1577
46	1.2136	0.1810	0.0313	0.9781	0.1066	13999.9303	0.0217	2.1702
47	1.7119	0.2623	0.0355	0.5375	0.0576	5469.3234	0.0217	2.1715

	dfbs	inf
1	0.0015	
2	0.0108	
3	-0.0584	
4	-0.0298	
5	0.0755	
6	-0.0001	
7	0.0137	
8	0.0500	
9	-0.0853	
10	0.1258	
11	-0.0268	
12	0.0390	
13	-0.0227	
14	0.1029	
15	-0.0087	
16	-0.0325	
17	0.0515	
18	0.0358	
19	0.0015	
20	0.0277	
21	-0.0312	
22	0.0155	
23	-0.0330	
24	-0.1416	
25	-0.0718	
26	0.0183	
27	-0.0575	
28	-0.0060	
29	-0.0298	
30	-0.0661	
31	-0.0094	
32	-0.0622	
33	-0.0641	
34	-0.0065	
35	-0.0576	
36	-0.0291	
37	0.0189	
38	-0.0099	
39	-0.0707	
40	-0.0373	
41	0.0338	
42	0.0527	
43	0.0653	
44	-0.0299	
45	-0.0509	
46	0.1809	
47	0.2600	

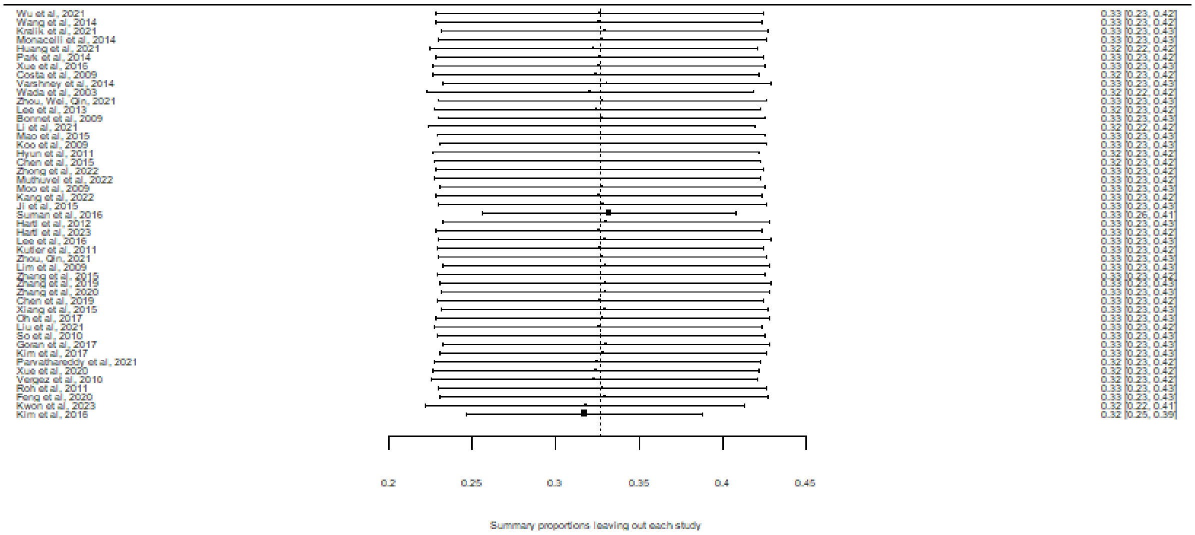

	estimate	se	zval	pval	ci.lb	ci.ub	Q	Qp	tau2
1	0.3265	0.0501	6.5217	0.0000	0.2284	0.4247	16292.4374	0.0000	0.1130
2	0.3261	0.0498	6.5417	0.0000	0.2284	0.4238	16294.0800	0.0000	0.1120
3	0.3295	0.0499	6.5978	0.0000	0.2316	0.4274	16291.0683	0.0000	0.1124
4	0.3281	0.0499	6.5765	0.0000	0.2303	0.4258	16296.2430	0.0000	0.1121
5	0.3229	0.0501	6.4488	0.0000	0.2247	0.4210	16202.0926	0.0000	0.1129
6	0.3266	0.0500	6.5376	0.0000	0.2287	0.4245	16294.1713	0.0000	0.1125
7	0.3259	0.0508	6.4167	0.0000	0.2264	0.4255	16264.9447	0.0000	0.1163
8	0.3241	0.0498	6.5139	0.0000	0.2266	0.4217	16292.2683	0.0000	0.1117
9	0.3308	0.0501	6.6095	0.0000	0.2327	0.4289	16267.4082	0.0000	0.1129
10	0.3204	0.0498	6.4310	0.0000	0.2228	0.4180	16186.9295	0.0000	0.1118
11	0.3279	0.0500	6.5641	0.0000	0.2300	0.4258	16296.3190	0.0000	0.1125
12	0.3247	0.0498	6.5179	0.0000	0.2270	0.4223	16290.2261	0.0000	0.1118
13	0.3277	0.0498	6.5799	0.0000	0.2301	0.4253	16296.4022	0.0000	0.1118
14	0.3215	0.0498	6.4543	0.0000	0.2239	0.4192	16252.2026	0.0000	0.1118
15	0.3270	0.0501	6.5251	0.0000	0.2288	0.4253	16294.8810	0.0000	0.1132
16	0.3282	0.0498	6.5946	0.0000	0.2307	0.4257	16296.3011	0.0000	0.1117
17	0.3241	0.0499	6.4990	0.0000	0.2263	0.4218	16280.5851	0.0000	0.1120
18	0.3248	0.0499	6.5136	0.0000	0.2271	0.4226	16286.7382	0.0000	0.1121
19	0.3265	0.0501	6.5209	0.0000	0.2284	0.4247	16292.3482	0.0000	0.1130
20	0.3252	0.0498	6.5260	0.0000	0.2276	0.4229	16291.2586	0.0000	0.1119
21	0.3281	0.0497	6.5980	0.0000	0.2307	0.4256	16296.3447	0.0000	0.1116
22	0.3258	0.0499	6.5325	0.0000	0.2281	0.4236	16292.2816	0.0000	0.1121
23	0.3282	0.0501	6.5468	0.0000	0.2300	0.4265	16295.4786	0.0000	0.1133
24	0.3320	0.0385	8.6240	0.0000	0.2565	0.4074	5184.0109	0.0000	0.0658
25	0.3301	0.0499	6.6140	0.0000	0.2323	0.4280	16287.1550	0.0000	0.1123
26	0.3257	0.0498	6.5466	0.0000	0.2282	0.4232	16295.3734	0.0000	0.1117
27	0.3295	0.0507	6.5009	0.0000	0.2301	0.4288	16269.8901	0.0000	0.1158
28	0.3269	0.0498	6.5684	0.0000	0.2294	0.4244	16296.2054	0.0000	0.1117
29	0.3281	0.0501	6.5439	0.0000	0.2298	0.4263	16295.9586	0.0000	0.1133
30	0.3299	0.0498	6.6227	0.0000	0.2322	0.4275	16293.5675	0.0000	0.1118
31	0.3271	0.0499	6.5601	0.0000	0.2293	0.4248	16296.0212	0.0000	0.1120
32	0.3297	0.0504	6.5409	0.0000	0.2309	0.4285	16272.5597	0.0000	0.1145
33	0.3298	0.0501	6.5871	0.0000	0.2317	0.4279	16284.1741	0.0000	0.1129
34	0.3269	0.0499	6.5578	0.0000	0.2292	0.4246	16295.8486	0.0000	0.1120
35	0.3294	0.0499	6.5965	0.0000	0.2316	0.4273	16291.1581	0.0000	0.1124
36	0.3281	0.0508	6.4590	0.0000	0.2285	0.4276	16295.3588	0.0000	0.1163
37	0.3257	0.0500	6.5116	0.0000	0.2276	0.4237	16286.3059	0.0000	0.1127

38	0.3271	0.0500	6.5407	0.0000	0.2291	0.4251	16295.5319	0.0000	0.1127
39	0.3301	0.0499	6.6196	0.0000	0.2324	0.4278	16290.1805	0.0000	0.1120
40	0.3284	0.0498	6.5922	0.0000	0.2308	0.4261	16296.0205	0.0000	0.1119
41	0.3249	0.0497	6.5334	0.0000	0.2275	0.4224	16294.7926	0.0000	0.1116
42	0.3240	0.0498	6.5090	0.0000	0.2264	0.4216	16290.4782	0.0000	0.1117
43	0.3234	0.0498	6.4928	0.0000	0.2258	0.4210	16283.2972	0.0000	0.1118
44	0.3281	0.0499	6.5805	0.0000	0.2304	0.4258	16296.2703	0.0000	0.1120
45	0.3291	0.0501	6.5740	0.0000	0.2310	0.4272	16290.8863	0.0000	0.1129
46	0.3179	0.0487	6.5303	0.0000	0.2225	0.4133	13999.9303	0.0000	0.1066
47	0.3173	0.0361	8.7937	0.0000	0.2466	0.3880	5469.3234	0.0000	0.0576

	I2	H2
1	99.7238	362.0542
2	99.7238	362.0907
3	99.7238	362.0237
4	99.7239	362.1387
5	99.7223	360.0465
6	99.7238	362.0927
7	99.7233	361.4432
8	99.7238	362.0504
9	99.7234	361.4980
10	99.7220	359.7095
11	99.7239	362.1404
12	99.7238	362.0050
13	99.7239	362.1423
14	99.7231	361.1601
15	99.7238	362.1085
16	99.7239	362.1400
17	99.7236	361.7908
18	99.7237	361.9275
19	99.7238	362.0522
20	99.7238	362.0280
21	99.7239	362.1410
22	99.7238	362.0507
23	99.7238	362.1217
24	99.1319	115.2002
25	99.7237	361.9368
26	99.7238	362.1194
27	99.7234	361.5531
28	99.7239	362.1379
29	99.7239	362.1324
30	99.7238	362.0793
31	99.7239	362.1338
32	99.7235	361.6124
33	99.7237	361.8705
34	99.7239	362.1300
35	99.7238	362.0257
36	99.7238	362.1191
37	99.7237	361.9179
38	99.7239	362.1229
39	99.7238	362.0040
40	99.7239	362.1338
41	99.7238	362.1065
42	99.7238	362.0106
43	99.7236	361.8510
44	99.7239	362.1393
45	99.7238	362.0197
46	99.6786	311.1096
47	99.1772	121.5405

1-2cm

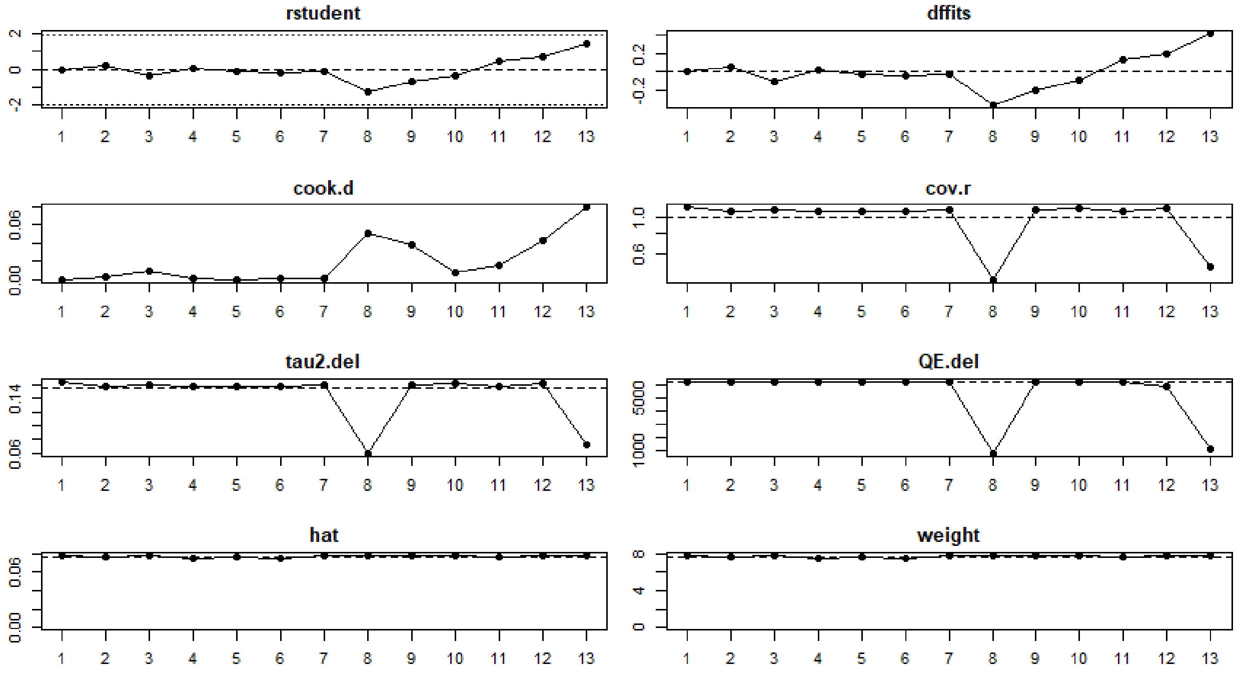

	rstudent	dffits	cook.d	cov.r	tau2.del	QE.del	hat	weight	dfbs	inf
1	-0.0024	-0.0007	0.0000	1.1443	0.1652	6292.3141	0.0778	7.7794	-0.0007	
2	0.2049	0.0590	0.0035	1.0936	0.1579	6299.6025	0.0765	7.6528	0.0590	
3	-0.3412	-0.0988	0.0100	1.1100	0.1602	6310.4223	0.0773	7.7349	-0.0988	
4	0.0720	0.0206	0.0004	1.0880	0.1573	6307.3667	0.0753	7.5329	0.0206	
5	-0.0592	-0.0170	0.0003	1.0929	0.1579	6308.6725	0.0763	7.6253	-0.0170	
6	-0.1545	-0.0438	0.0019	1.0855	0.1571	6310.3779	0.0743	7.4326	-0.0438	
7	-0.0917	-0.0265	0.0007	1.1033	0.1593	6307.8732	0.0771	7.7124	-0.0265	
8	-1.2193	-0.3652	0.0508	0.4241	0.0596	800.7390	0.0781	7.8119	-0.3609	
9	-0.6697	-0.1940	0.0385	1.1106	0.1603	6295.1433	0.0774	7.7429	-0.1940	
10	-0.3059	-0.0887	0.0081	1.1203	0.1617	6310.6489	0.0776	7.7559	-0.0887	
11	0.4432	0.1273	0.0163	1.0892	0.1574	6291.1316	0.0761	7.6140	0.1272	
12	0.7043	0.2047	0.0436	1.1282	0.1628	5882.4233	0.0780	7.7950	0.2048	
13	1.4112	0.4173	0.0795	0.5047	0.0714	1082.9778	0.0781	7.8101	0.4137	

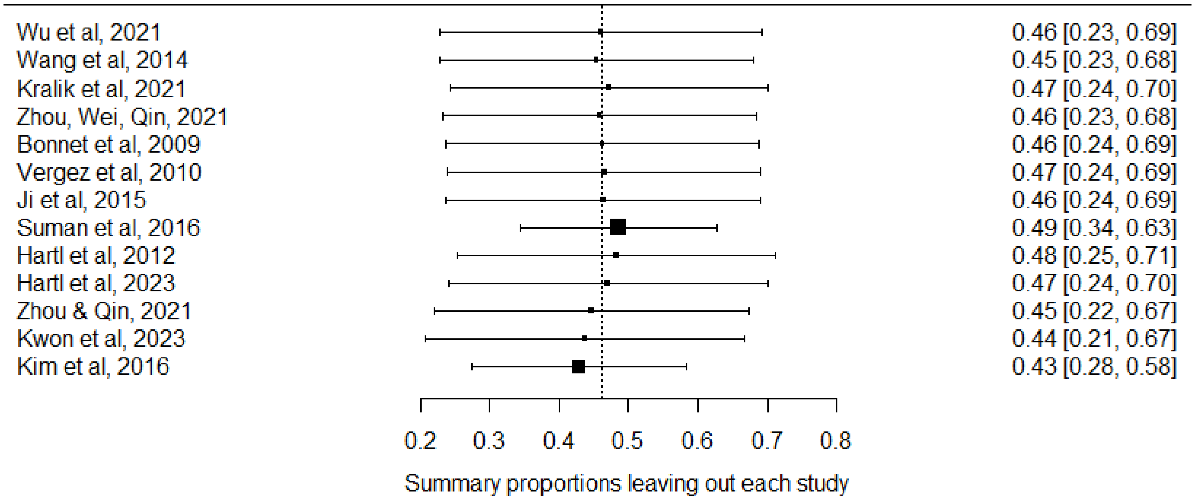

	estimate	se	zval	pval	ci.lb	ci.ub	Q	Qp	tau2
1	0.4605	0.1182	3.8944	0.0001	0.2287	0.6922	6292.3141	0.0000	0.1652
2	0.4538	0.1156	3.9264	0.0001	0.2273	0.6804	6299.6025	0.0000	0.1579
3	0.4714	0.1165	4.0483	0.0001	0.2432	0.6997	6310.4223	0.0000	0.1602
4	0.4581	0.1153	3.9734	0.0001	0.2321	0.6841	6307.3667	0.0000	0.1573
5	0.4623	0.1156	4.0006	0.0001	0.2358	0.6888	6308.6725	0.0000	0.1579
6	0.4653	0.1152	4.0400	0.0001	0.2395	0.6910	6310.3779	0.0000	0.1571
7	0.4634	0.1161	3.9909	0.0001	0.2358	0.6909	6307.8732	0.0000	0.1593
8	0.4853	0.0720	6.7419	0.0000	0.3442	0.6264	800.7390	0.0000	0.0596
9	0.4821	0.1165	4.1388	0.0000	0.2538	0.7104	6295.1433	0.0000	0.1603
10	0.4704	0.1170	4.0204	0.0001	0.2411	0.6997	6310.6489	0.0000	0.1617
11	0.4463	0.1154	3.8688	0.0001	0.2202	0.6724	6291.1316	0.0000	0.1574
12	0.4373	0.1174	3.7248	0.0002	0.2072	0.6674	5882.4233	0.0000	0.1628
13	0.4292	0.0785	5.4663	0.0000	0.2753	0.5831	1082.9778	0.0000	0.0714

	I2	H2
1	99.8252	572.0286
2	99.8254	572.6911
3	99.8257	573.6748
4	99.8256	573.3970
5	99.8256	573.5157
6	99.8257	573.6707
7	99.8256	573.4430
8	98.6263	72.7945
9	99.8253	572.2858
10	99.8257	573.6954
11	99.8252	571.9211
12	99.8130	534.7658
13	98.9843	98.4525

2-4cm

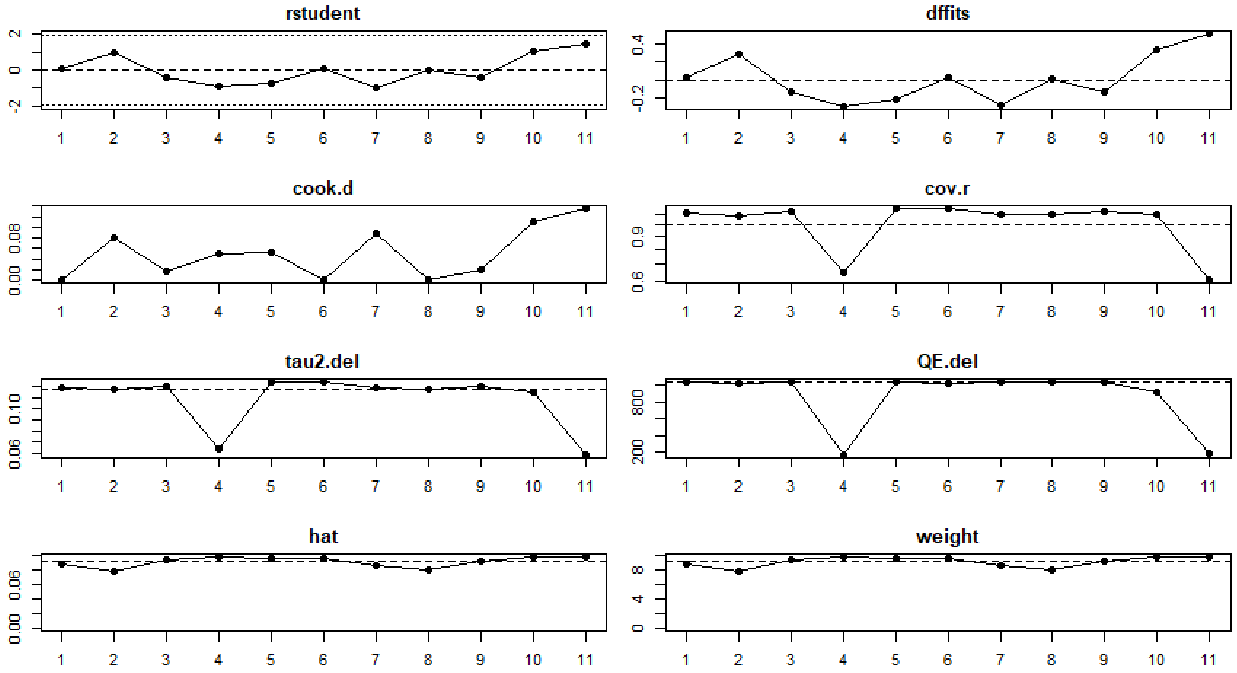

	rstudent	dffits	cook.d	cov.r	tau2.del	QE.del	hat	weight	dfbs
1	0.0903	0.0279	0.0008	1.1090	0.1184	1032.4457	0.0876	8.7615	0.0279
2	0.9740	0.2821	0.0795	1.0834	0.1168	1026.5953	0.0774	7.7407	0.2821
3	-0.4093	-0.1312	0.0177	1.1335	0.1204	1034.4956	0.0930	9.3030	-0.1312
4	-0.8595	-0.2990	0.0490	0.6469	0.0641	172.4660	0.0986	9.8574	-0.2909
5	-0.6921	-0.2248	0.0532	1.1623	0.1233	1032.1467	0.0956	9.5578	-0.2250
6	0.0660	0.0211	0.0005	1.1661	0.1237	1025.9626	0.0960	9.5986	0.0211
7	-0.9615	-0.2941	0.0873	1.1037	0.1180	1032.3283	0.0854	8.5442	-0.2940
8	0.0145	0.0042	0.0000	1.0944	0.1177	1033.7075	0.0799	7.9856	0.0042
9	-0.4478	-0.1419	0.0205	1.1225	0.1194	1034.4825	0.0910	9.1032	-0.1419
10	1.0179	0.3342	0.1102	1.0937	0.1153	906.2757	0.0971	9.7131	0.3341
11	1.4630	0.5176	0.1363	0.6044	0.0593	191.3488	0.0983	9.8349	0.5019

	inf
1	
2	
3	
4	
5	
6	
7	
8	
9	
10	
11	

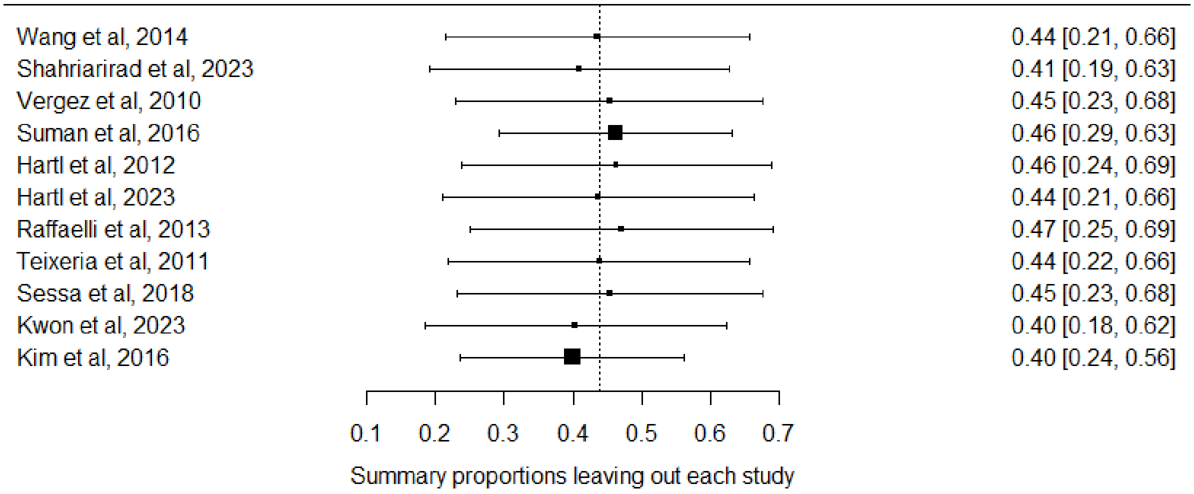

	estimate	se	zval	pval	ci.lb	ci.ub	Q	Qp	tau2
1	0.4361	0.1130	3.8579	0.0001	0.2146	0.6577	1032.4457	0.0000	0.1184
2	0.4089	0.1117	3.6593	0.0003	0.1899	0.6278	1026.5953	0.0000	0.1168
3	0.4534	0.1143	3.9674	0.0001	0.2294	0.6774	1034.4956	0.0000	0.1204
4	0.4629	0.0863	5.3614	0.0000	0.2937	0.6321	172.4660	0.0000	0.0641
5	0.4639	0.1157	4.0086	0.0001	0.2371	0.6907	1032.1467	0.0000	0.1233
6	0.4368	0.1159	3.7682	0.0002	0.2096	0.6640	1025.9626	0.0000	0.1237
7	0.4708	0.1128	4.1752	0.0000	0.2498	0.6919	1032.3283	0.0000	0.1180
8	0.4387	0.1123	3.9063	0.0001	0.2186	0.6588	1033.7075	0.0000	0.1177
9	0.4545	0.1137	3.9964	0.0001	0.2316	0.6774	1034.4825	0.0000	0.1194
10	0.4035	0.1123	3.5941	0.0003	0.1835	0.6235	906.2757	0.0000	0.1153
11	0.3995	0.0835	4.7872	0.0000	0.2359	0.5631	191.3488	0.0000	0.0593

	I2	H2
1	99.1283	114.7162
2	99.1233	114.0661
3	99.1300	114.9440
4	94.7816	19.1629
5	99.1280	114.6830
6	99.1228	113.9958
7	99.1282	114.7031
8	99.1293	114.8564
9	99.1300	114.9425
10	99.0069	100.6973
11	95.2965	21.2610

Greater than 4cm

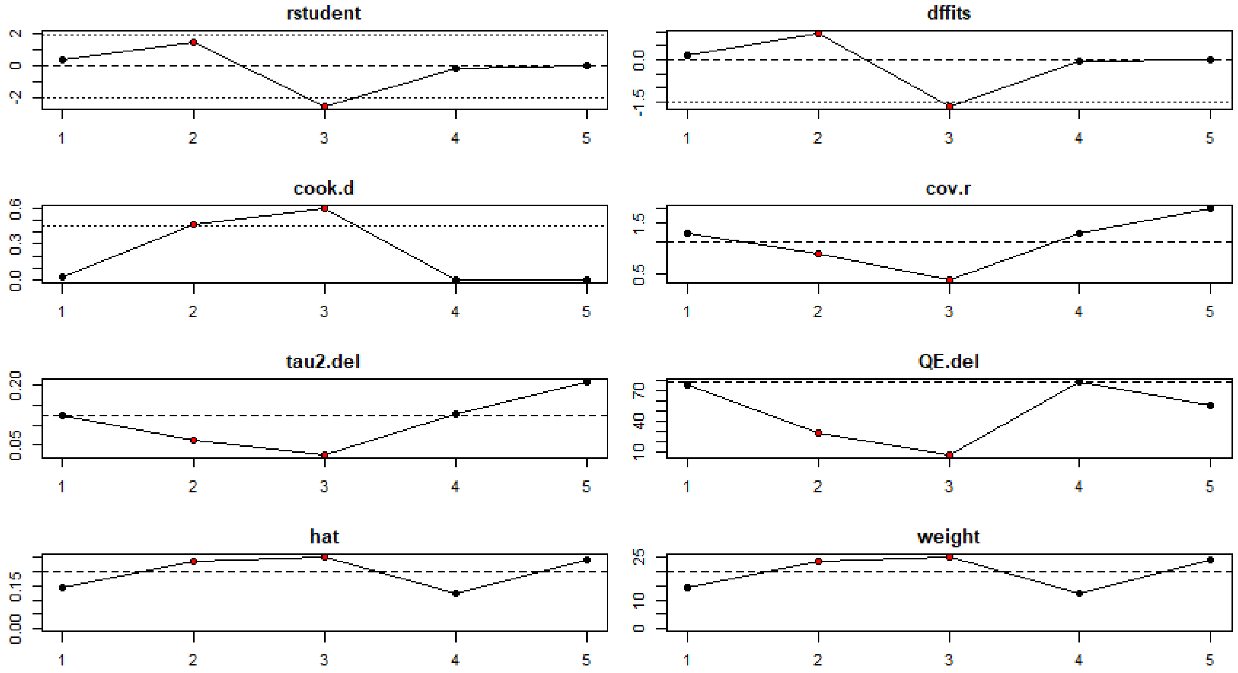


```
rstudent   dffits  cook.d   cov.r tau2.del   QE.del     hat   weight     dfbs
1    0.4039   0.1652 0.0274 1.1763    0.1236 75.9652 0.1435 14.3452   0.1650
2    1.4345   0.9190 0.4629 0.7656    0.0631 28.0583 0.2359 23.5851   0.8965
3   -2.4747  -1.6358 0.5989 0.4422    0.0273  7.5405 0.2529 25.2907  -1.4251
4   -0.1622  -0.0640 0.0042 1.1756    0.1264 78.2808 0.1253 12.5345  -0.0637
5   -0.0064  -0.0242 0.0010 2.0355    0.2064 55.8292 0.2424 24.2445  -0.0248
  inf
1
2   *
3   *
4
5
```

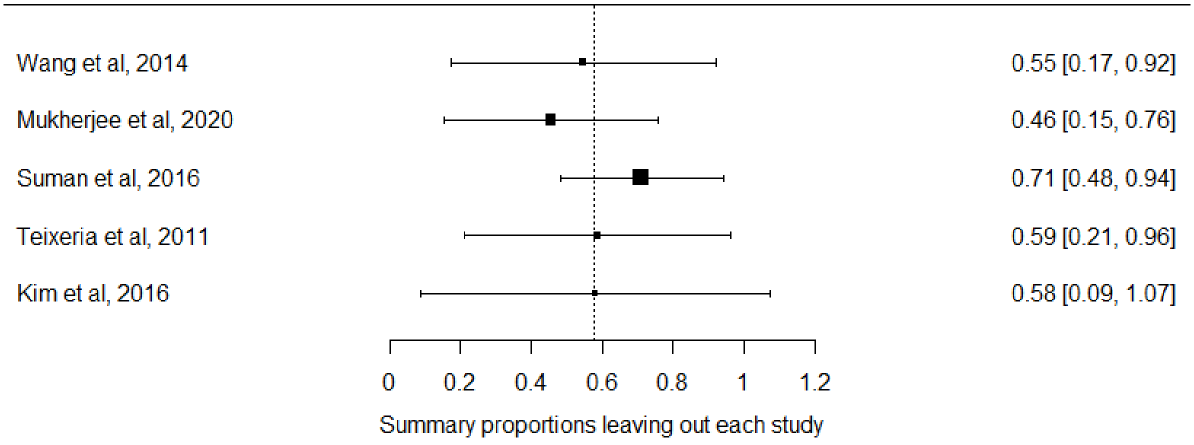

	estimate	se	zval	pval	ci.lb	ci.ub	Q	Qp	tau2
1	0.5465	0.1911	2.8603	0.0042	0.1720	0.9210	75.9652	0.0000	0.1236
2	0.4558	0.1541	2.9570	0.0031	0.1537	0.7579	28.0583	0.0000	0.0631
3	0.7120	0.1171	6.0779	0.0000	0.4824	0.9416	7.5405	0.0565	0.0273
4	0.5870	0.1910	3.0734	0.0021	0.2127	0.9614	78.2808	0.0000	0.1264
5	0.5811	0.2513	2.3122	0.0208	0.0885	1.0737	55.8292	0.0000	0.2064

	I2	H2
1	96.0508	25.3217
2	89.3080	9.3528
3	60.2150	2.5135
4	96.1676	26.0936
5	94.6265	18.6097
