# Supplementary material for: Prevalence of Occult Central Lymph Node Metastasis by Tumor Size in Papillary Thyroid Carcinoma: A Systematic Review and Meta-Analysis
Source: Curr Oncol. 2023 Aug 2;30(8):7335–50. doi: 10.3390/curroncol30080532 (PMC10453273; doi:10.3390/curroncol30080532)
Supplement: Supplementary file 1 [file curroncol-30-00532-s001.zip › Supplemental Figure 2 Funnel Egger.pdf]

Supplemental Figure S2.
Funnel plots and Egger publication bias statistics outputs

<u>Less than 5mm</u>

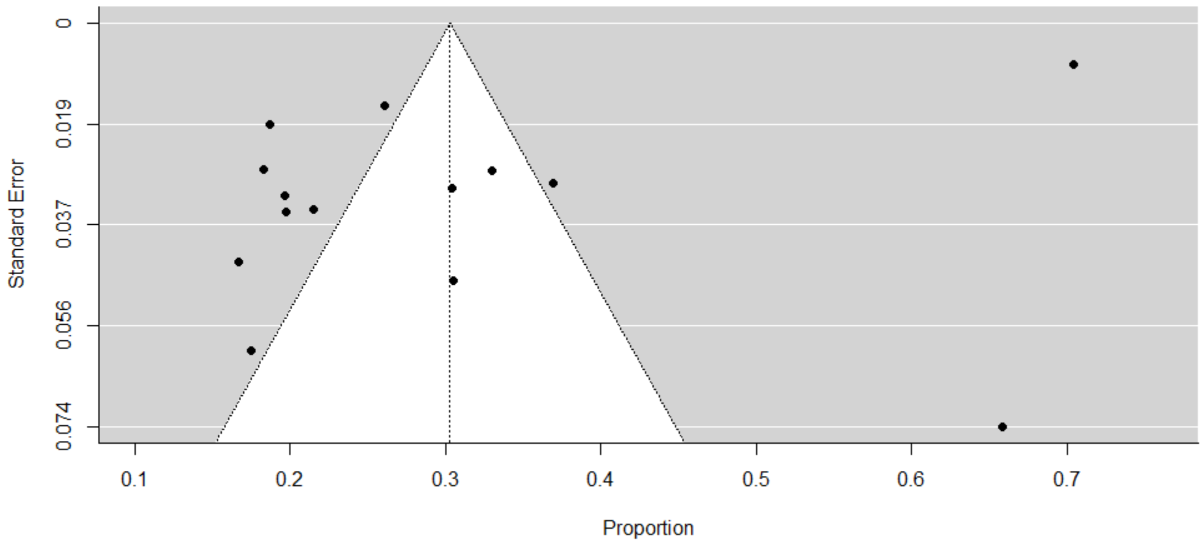


```
Regression Test for Funnel Plot Asymmetry

Model:     mixed-effects meta-regression model
Predictor: standard error

Test for Funnel Plot Asymmetry: z = 0.0719, p = 0.9427
Limit Estimate (as sei -> 0):   b = 0.2938 (CI: 0.0274, 0.5603)
```

Less than 1cm

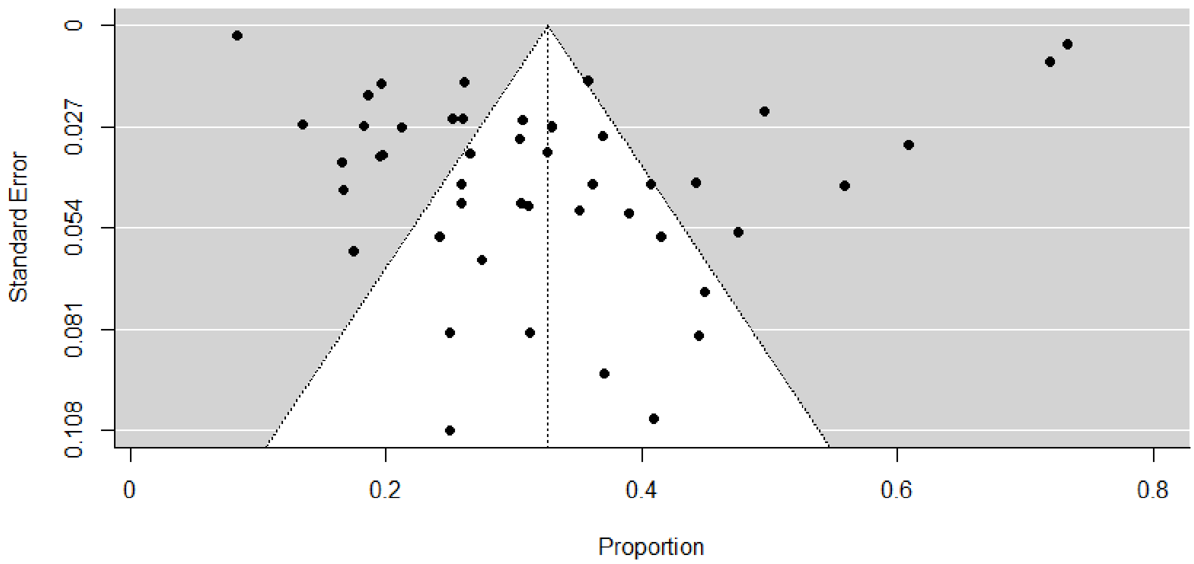


```
Regression Test for Funnel Plot Asymmetry

Model:     mixed-effects meta-regression model
Predictor: standard error

Test for Funnel Plot Asymmetry: z = 0.0106, p = 0.9915
Limit Estimate (as sei -> 0):   b = 0.3256 (CI: 0.1206, 0.5307)
```

1-2cm

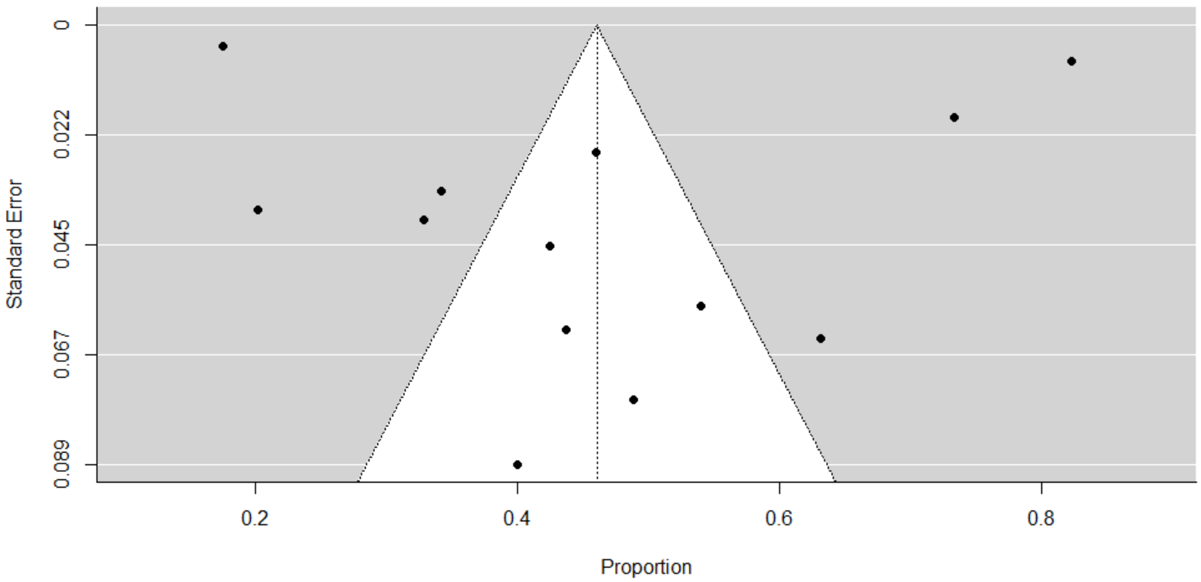


```
Regression Test for Funnel Plot Asymmetry

Model:     mixed-effects meta-regression model
Predictor: standard error

Test for Funnel Plot Asymmetry: z = -0.0861, p = 0.9314
Limit Estimate (as sei -> 0):   b =  0.4776 (CI: 0.0261, 0.9290)
```

2-4cm

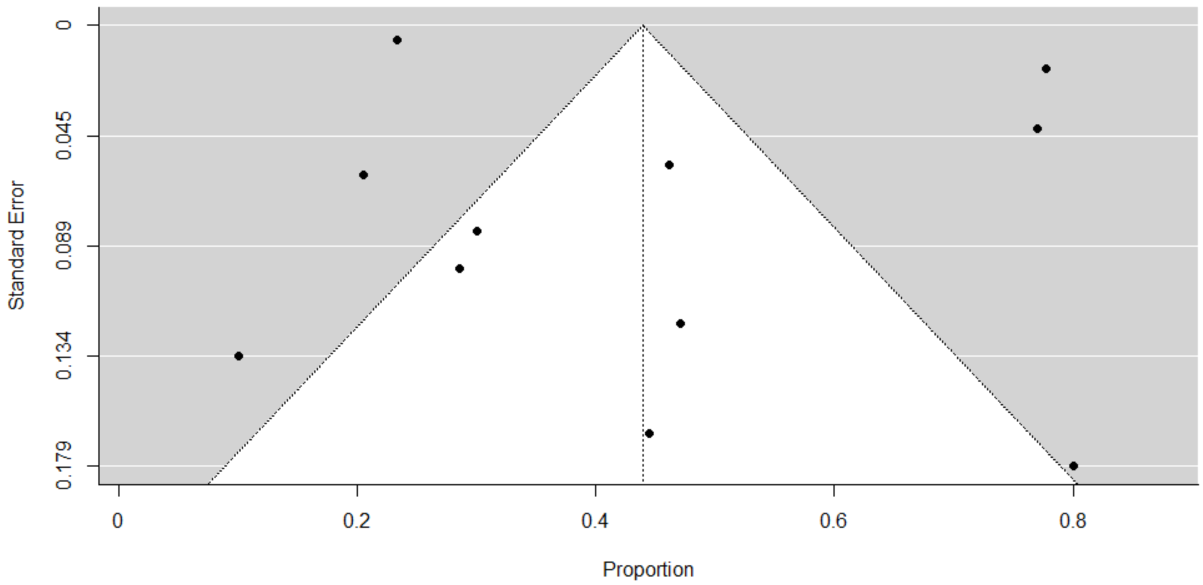


```
Regression Test for Funnel Plot Asymmetry

Model:     mixed-effects meta-regression model
Predictor: standard error

Test for Funnel Plot Asymmetry: z = -0.0544, p = 0.9566
Limit Estimate (as sei -> 0):   b =  0.4488 (CI: 0.0393, 0.8582)
```

Greater than 4cm

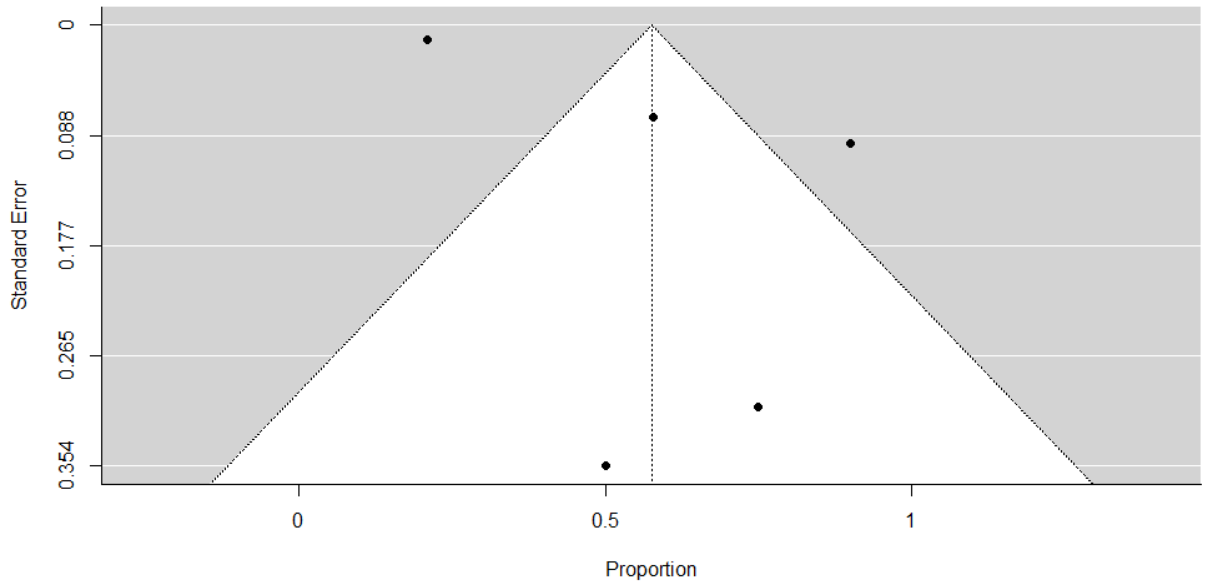


```
Regression Test for Funnel Plot Asymmetry

Model:     mixed-effects meta-regression model
Predictor: standard error

Test for Funnel Plot Asymmetry: z = 0.6351, p = 0.5253
Limit Estimate (as sei -> 0):   b = 0.4752 (CI: 0.0662, 0.8841)
```
